# FEM Analysis of Textile Reinforced Composite Materials Impact Behavior

**DOI:** 10.3390/ma14237380

**Published:** 2021-12-02

**Authors:** Savin Dorin Ionesi, Luminita Ciobanu, Catalin Dumitras, Manuela Avadanei, Ionut Dulgheriu, Irina Ionescu, Maria Carmen Loghin

**Affiliations:** 1 Faculty of Industrial Design and Business Management, “Gheorghe Asachi” Technical University of Iasi, Blvd. Mangeron, No. 29, 700050 Iasi, Romania; mavad@tuiasi.ro (M.A.); idulgheriu@tuiasi.ro (I.D.); iirina@tuiasi.ro (I.I.); 2 Faculty of Machine Manufacturing and Industrial Management, “Gheorghe Asachi” Technical University of Iasi, Blvd. Mangeron, No. 59, 700050 Iasi, Romania; dumitrascata@yahoo.com

**Keywords:** FEM, impact behavior, composite materials, knitted fabrics

## Abstract

Composite materials reinforced with textile fabrics represent a complex subject. When explaining these materials, one must consider their mechanical behavior in general, and impact resistance in particular, as many applications are characterized by dynamic strains. Impact characteristics must be considered from the early stages of the design process in order to be controlled through structure, layer deposition and direction. Reinforcement materials are essential for the quality and behavior of composites, and textile reinforcements present a large range of advantages. It takes a good understanding of the requirements specific to an application to accurately design textile reinforcements. Currently, simulations of textile reinforcements and composites are efficient tools to forecast their behavior during both processing and use. The paper presents the steps that must be followed for modelling the impact behavior of composite materials, using finite element analysis (FEM). The FEM model built using Deform 3D software offers information concerning the behavior structure during impact. The behavior can be visualized for the structure as a whole and, for different sections, be considered significant. Furthermore, the structure’s strain can be visualized at any moment. In real impact tests, this is not possible due to the very short time interval and the impossibility to record inside the structure, as well as to record all significant stages using conventional means.

## 1. Introduction

Mechanical properties of textile reinforced composites are essential in applications characterized by a high level of strains, and predicting their behavior during use is a determinant for the design stage and ensures its quality. The complexity of textile structures and geometries, their anisotropy and discontinuous nature make the process of modelling mechanical behavior extremely difficult.

In terms of prediction, applied mathematics is used to define boundary conditions in solving continuum mechanics. The scientific literature contains studies of applied physics aimed at solving similar problems, but is directed to obtain continuous functions based on approximated areas. Aerospace engineering researches focus on finding optimal ways of expressing the influence of the rigidity coefficients. All these results can be summed up in three distinct approaches and solutions, each expressing a different view on the same issue, namely modelling the behavior of composite materials.

The term finite element method (FEM) defines a broad range of computing techniques that have certain common characteristics. Currently the main drawbacks related to application difficulties have been removed with the widespread use of computers and the development of a large range of analytic software such as ANSYS, Algor or Deform 3D.

In the textile domain, modelling using the finite element method has experienced an accelerated growth in the last decade, most models predicting the mechanical behavior of composite materials with woven reinforcement [1,2,3,4,5].

Even if they have a lower mechanical resistance, knitted fabrics are widely used for composite reinforcement. The complexity of their specific geometry and the large number of structural possibilities, as well as the direct influence of the mechanical strains introduced during knitting at yarn level, led to the formulation of different geometrical and mechanical models for knitted fabrics, each one based on different assumptions and production conditions, and considered the micro, meso or macro level of the fabrics. Mechanical models of knitted fabrics can be divided into models based on force analysis, models based on energy minimization, and FEM models [6].

The situation is even more complicated in the case of knitted fabrics built along all the three axes, the so-called 3D knitted fabrics, as it is not only the fabric structure that has to be modelled, but also the 3D geometry of that fabric and its particularities in connection between stitches.

This is the case of weft/warp knitted spacer fabrics, materials characterized by the presence of (at least) two independent layers connected by yarns or by knitted layers [7,8]. Connection through yarns limits the spatial geometry of the 3D fabrics but is widely used in technical applications, especially as warp knitted fabrics. A large range of possibilities is offered by the flexibility of weft knitted spacers with connections through knitted layers [9,10,11,12]. Such possibilities are beneficial for developing preforms for composite materials with the final shape of the product.

In the literature, there are fewer FEM models for the mechanical behavior of knitted fabrics mainly due to the complexity of the yarn geometry in the stitches and the high number of factors influencing their properties.

All FEM models are based on geometrical models developed for knitted fabrics in general, there is an exhaustive review being carried out in this [13], or for knitted fabrics for composite reinforcement, considering the particularities of high-performance fibers [14,15,16]. These models concern mainly 2D knitted fabrics. Still, a literature survey shows a limited number of models dealing with the mechanical behavior of warp knitted spacers exemplified by [17,18,19], as well as weft knitted spacers [20,21]. The model proposed by Hamedi [21], simulates the flexural behavior of weft knitted spacer fabrics with a constant width (connected through knitted layers), considering the geometrical specificities proposed by Vassiliadis [22].

The paper presents a different approach for the modelling of mechanical behavior of composite materials reinforced with weft knitted spacer fabrics, by replacing the composite with an equivalent continuous material, with similar mechanical characteristics. The advantage of such an equivalence is given by the elimination of the numerical models for the knitted structure with all their restrictions and limitations. Furthermore, an equivalent continuous material can be placed easily to form the required specific geometry. This is an innovative approach to modelling composites reinforced with knitted fabrics that simplifies the behavior simulation. The approach requires careful consideration in determining the characteristics of the equivalent continuous material to correspond to the properties of real composite.

The equivalent model proposed in the paper deals with low velocity impact behavior of a composite material reinforced with knitted spacers of predetermined geometry (distance between connecting layers and distance between independent layers). The proposed model is described and used to predict impact behavior. Validation is carried out based on experimental results to see the level of conformity the model presents.

## 2. Materials and Methods

**Figure 1:**
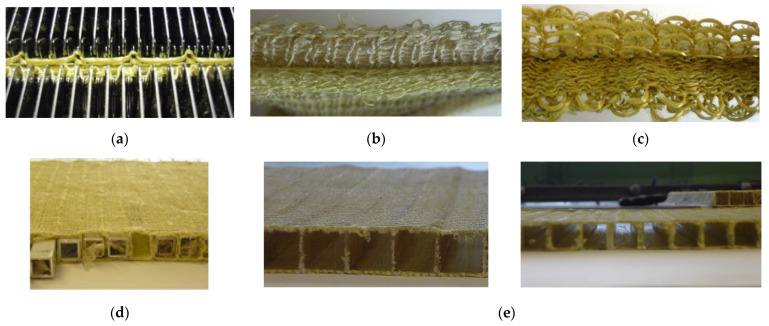
Composite reinforced with sandwich knitted fabrics—machine view (**a**), aspect of the outer layers with (**b**) and without in-lay yarns (**c**), preparation of the knitted reinforcement–insertion of moulds (**d**) and final aspect of the knitted spacer reinforced composite (**e**).

The knitted reinforcement selected for this study is a sandwich fabric where the independent outer layers (plain jersey) are connected through knitted layers placed perpendicularly to them. The distance between the connecting layers is 10 mm. The samples were designed without or with inlay yarns in the exterior layers (yarns placed horizontally in the fabric, without looping it into stitches). Inlay yarns are an efficient way to enhance fabric strength. Figure 1 presents the production of the textile reinforced composite, starting with the knitting of the reinforcement.

The 3D knitted fabric variants were produced on a STOLL CMS 320 TC flat knitting machine, Reutlingen, Germany, gauge 10E. The fabrics were made of para-aramid (Steel Kevlar and Twaron, DuPont, Wilmington, DE, USA) and technical natural yarns (Linen). The use of natural fibers was designed so as not to affect the performance of the spacer fabrics, and target an increase in sustainability. Different raw materials were used to produce the outer and connecting layers, as presented in Table 1. The volume fraction of the composite samples required to increase fabric compactness was obtained by introducing transversal Twaron yarns.

The 3D composite materials studied in this paper were produced using the 4 variants of the spacer fabrics presented in Table 1 as performs, and epoxy EPICURE 04908, Hexion, Columbus, OH, USA, and polyester DISTITRON 3501S, Polynt, Bergamo, Italy, as matrices, resulting in two sets of composites. The epoxy matrix had a mixing ratio of 30% EPIKURE Curing Agent 04908 and 5% Dearing agent BYK A535, while for the polyester resin the mixing contained 1.5% of initiator for unsaturated polyester resin NOROX MCP 75 and 0.08% polyester inhibitor NLC 10. The composite materials were processed using the Vacuum Assisted Resin Transfer Molding (VARTM) technology. All experimental samples were cured at room temperature (23 °C), the ones with epoxy matrix for 46 h and the ones with polyester for 23 h.

The modelling of the behavior to low intensity impact of the composite materials has to be based on the physical-mechanical properties of the reinforcement and matrix used for composite materials. The experimental values obtained for these properties are shown in Table 2.

## 3. Definition of the Model

**Figure 2:**
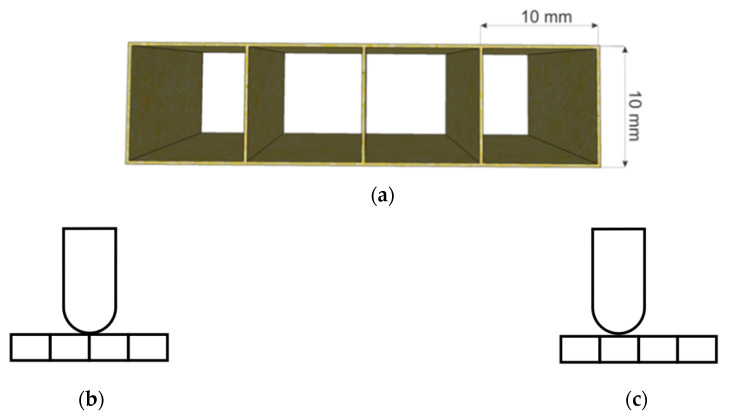

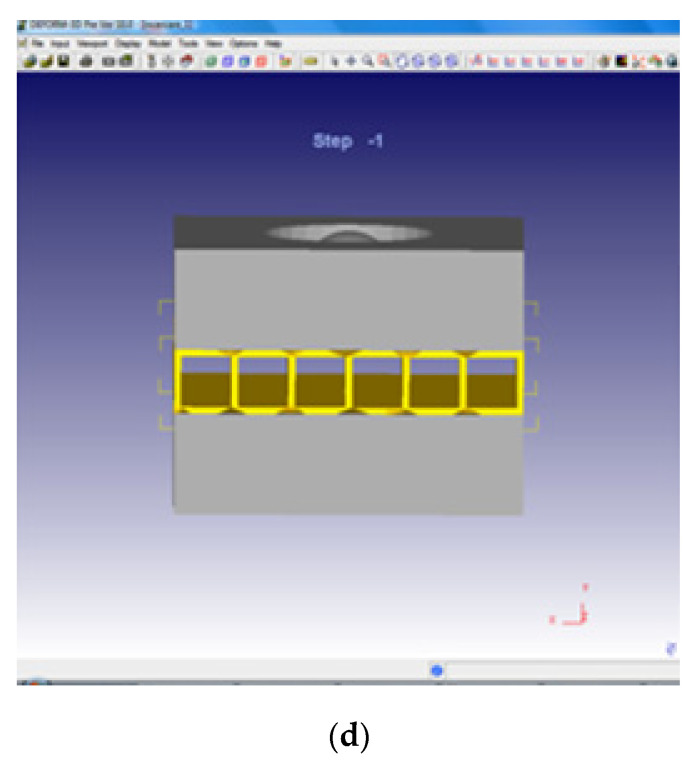
Model design. (**a**) Geometrical model for the equivalent continuous material. (**b**) Impactor position on connection layer. (**c**) Impactor position between connection layers. (**d**) Aspect of processed model.

The general geometry of the composite material to be analyzed using FEM is defined in Figure 2a, where the exterior walls and connecting layers form the repetitive element, the partition.

Taking into consideration the fact that a composite material is an advanced structure made from at least two distinct materials that are combined at a macroscopic scale with different mechanical properties, a composite structure with equivalent mechanical properties was considered.

Due to the specific 3D geometry of the composite, the equivalent continuous model presents open areas with predefined dimensions, and, therefore, it is important to select the position of the impactor used for low velocity impact test simulations in relation to the connecting walls, as illustrated in Figure 2. We estimated that two positions are of consequence: one illustrated in Figure 2b shows that the impactor is placed on the connecting layers, while in Figure 2c the impactor is placed between connecting layers. These two positions exemplify the possibilities of impact during composite’s life cycle.

The model was designed in a DEFORM 3D software, DEFORM, Columbus, OH, USA, application and was meshed with “brick” or “tetrahedral” elements [23], as illustrated in Figure 2d. The mesh size was adjusted from fine to coarse and the number of finite elements was established at 180,000/volume.

The main steps to be taken in order to perform a finite element analysis can be grouped into a number of operations, as follows:

- Division into finite elements (discretization);
- Defining interpolation functions;
- Defining the equations of finite elements;
- Assembling elementary equations into system equations;
- Solving the obtained system of equations;
- Performing additional calculations if necessary to determine secondary unknowns.

For model calibration an initial testing was carried out on similar composite materials. A Fractovis Plus impact testing machine, Instron, Norwood, MA, USA, equipped with a round head impactor and pneumatic clamping system was used to carry out the tests.

**Figure 3:**
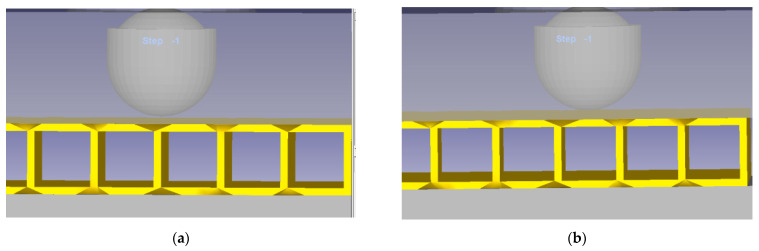
Model of impactor head and composite structure. (**a**) On connection layer. (**b**) Between connection layers.

**Figure 4:**
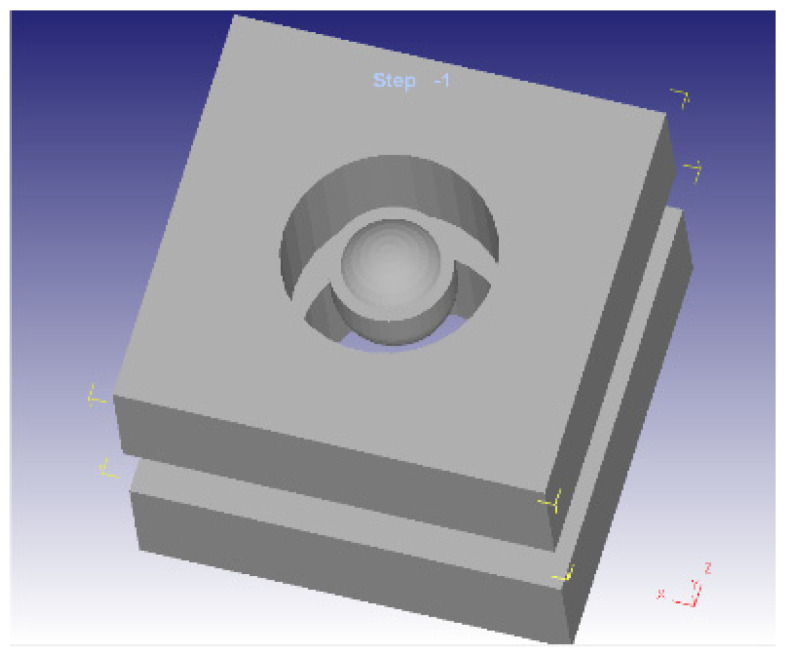
Mode of impactor head and structure of clamping plates.

All physical pieces involved in the impact testing were modelled. The impactor position on connection layer is presented in Figure 3a, while impactor position between connection layers is presented in Figure 3b. The model of impactor head and structure of clamping plates is presented in Figure 4. The loading force and the processing speed were experimentally determined based on the captured data using the Fractovis Plus impact testing machine, illustrated in Table 3.

## 4. Results and Discussions

### 4.1. Simulation of the Deformation Process

**Figure 5:**
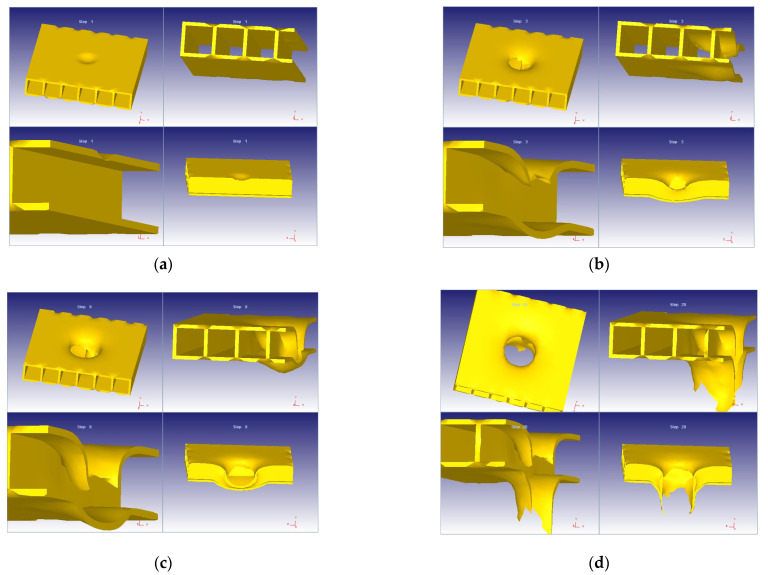
Impactor action. (**a**) Step 1. (**b**) Step 3. (**c**) Step 8. (**d**) Step 20.

The deformation of the model was processed using DEFORM 3D. The simulation results in different steps of the action of the impactor on the 3D composite material is illustrated in Figure 5a–d.

**Figure 6:**
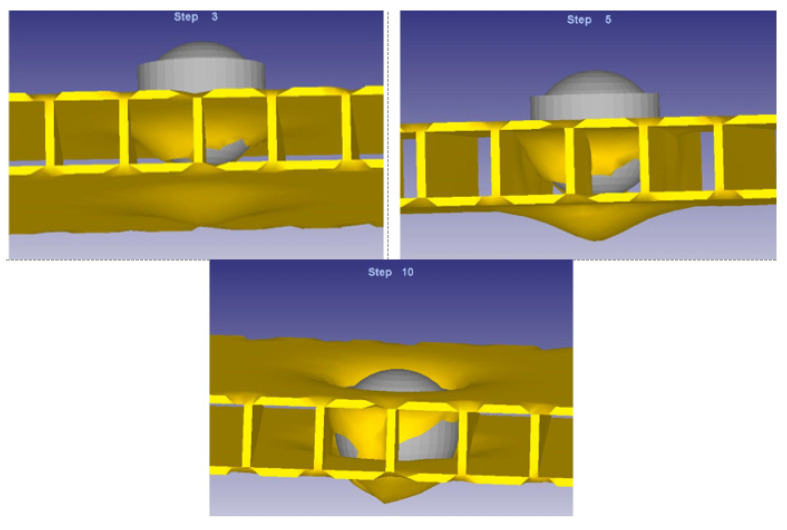
Impactor head action.

It should be noted that in the previous figures not all the elements participating in the process appear the way the impactor head acts, being presented in Figure 6.

**Figure 7:**
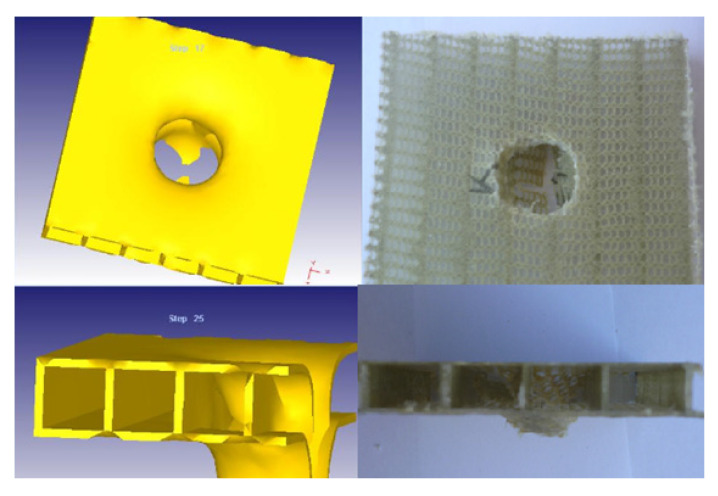
Comparison of the breaking area for the simulated model and impacted samples.

The simulation results were compared with those obtained after performing a real experiment, as illustrated in Figure 7.

The program provides information on the time frames for each deformation snapshot, and about the characteristics of the process in that moment (work speed, force deformation, the value of the impactor stroke, etc.), as illustrated in Table 4.

**Figure 8:**
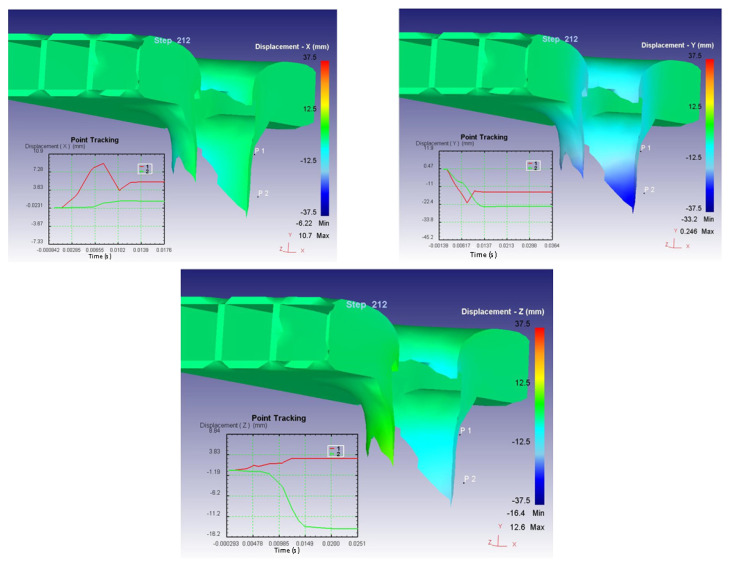
Variation on the 3 axes of the displacement field.

Figure 8 present the variation on the three axes of the displacement field on the composite structure following the impact.

The curves in the graphs, illustrated in Figure 8, represent the evolution of the displacements in the direction of the specified axes in two points, considered P1 and P2, arranged on the surfaces (point 1 is arranged on the upper surface and point 2 on the lower surface). On the right side is the color legend of the displacements expressed in mm.

**Figure 9:**
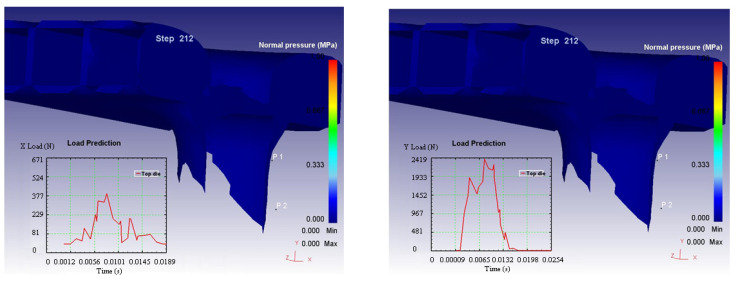

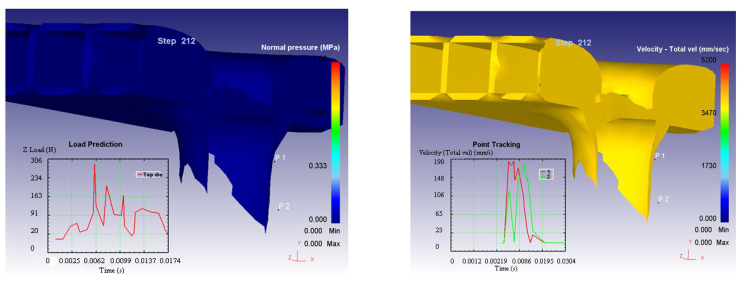
Variation on the 3 axes of the strain force and velocity.

Figure 9 presents the variation on the three axes of the strain forces on the composite structure following the impact.

Finite element modelling and analysis is particularly important in the context of the research conducted because it provides an impressive amount of information regarding the studied process, namely the impact stress of the composite structure reinforced with textile yarns. However, this information may be of no value if it is not continued and verified with one or more experimental tests performed under conditions similar to those in which the analysis was performed. These experiments will validate the finite element analysis, and once it has validated several values (e.g., the level of forces in the system, working speeds, process dynamics, etc.), it is assumed that all values, and implicitly the simulations, have been validated.

### 4.2. Validation—Experimental Results

The results from the simulation were compared to the results obtained for the composite materials reinforced with spacer weft knitted U-shaped structures defined in Table 1.

**Figure 10:**
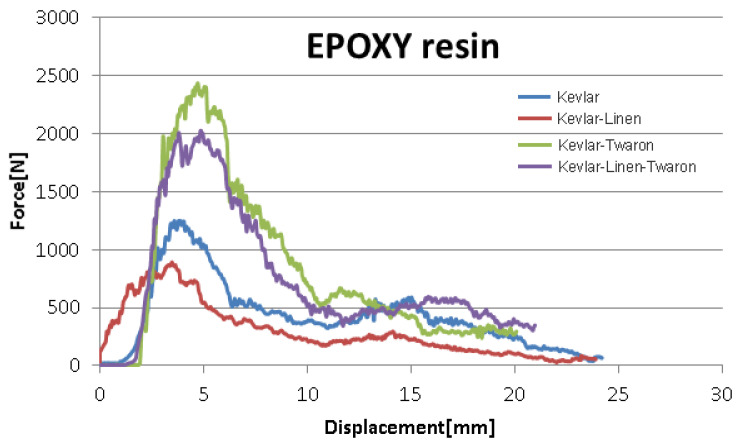
Force displacement curves—EPOXY resin.

**Figure 11:**
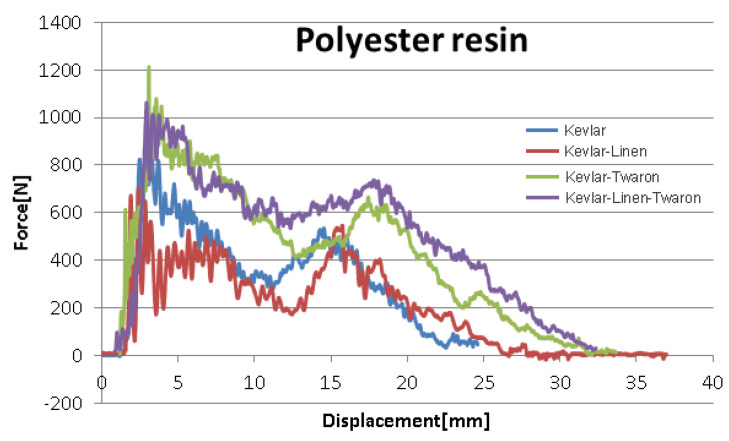
Force displacement curves—Polyester resin.

Considering the results of the experimental low velocity impact tests for the samples of composite materials defined in Table 1, it can be stated that the simulation with finite elements was confirmed because the level of forces in the system (shown in the figures above) at the simulation level corresponds as an order of magnitude, size, and approaches as a value, the level experimentally determined. Thus, it is observed that we have as an order of magnitude values of thousands of N (more precisely between 1200 and 2480 N, as illustrated in Figure 10 and Figure 11) for the force measured at an experimental level, and variations from 1276 N to 2781 N in the case of the simulation performed.

Furthermore, the experimental results show that the maximum level of displacement is placed between 10 and 36.56 mm, depending on the structure and the material used. In the case of the simulation, the values for virtual displacement vary between 27 and 37 mm.

It is observed that the intervals of variation of the forces in the system, and of the displacements obtained experimentally and virtually are significantly close in order of magnitude, and values which lead to the conclusion that the way the data were introduced and the conditions imposed to obtain process simulation are correct. Existing differences can be attributed to the characteristics imposed to the equivalent continuous material used for the simulation, and these characteristics can be corrected in order to improve the accuracy of the simulation.

Based on this, it can be stated that the simulation performed is confirmed by the experimental results.

### 4.3. Comparative Analysis

The information provided by the simulation is more detailed than the experiment itself. This information can be broken down into several areas.

A first information refers to the behavior of the structure during the impact stress. This behavior can be viewed on the simulated structure in its entirety and, in different sections, be considered relevant. Moreover, it can be seen how the structure is deformed at different moments. This is not possible in reality because the time interval in which the process takes place is very short, while the means of recording the image do not allow access inside the structure. Furthermore, the short time in which the process takes place leads to the impossibility of recording all the important phases by classical means.

From the deformation of the composite structure, two cases can be identified in which the process takes place, depending on the position between the impactor head and the composite structure:

- Case 1 results from the position of the axis of symmetry of the impactor head in relation to the partition. In this situation the impactor head passes through the partition wall (defined in Figure 2b).
- Case 2, the axis of symmetry of the impactor head passes between the partitions (defined in Figure 2c).

**Figure 12:**
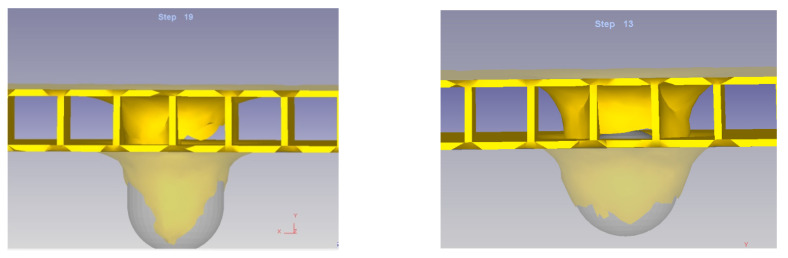
Deformations of the structure in the two considered cases.

The deformations of the structure in the two considered cases are illustrated in Figure 12.

It can be remarked that the penetration time in the second case is shorter and also the deformations are different in size. This phenomenon leads to the idea that the level of forces of the unitary efforts are different in the two situations.

In addition to data on the deformed structure (which can be presented as a “record” of the event), the simulation provides very important information on the field of displacement, deformation, unit effort, as well as the deformation forces in the system at any given time during the process presented in the form of a diagram. This phenomenon has been explicitly presented previously.

**Figure 13:**
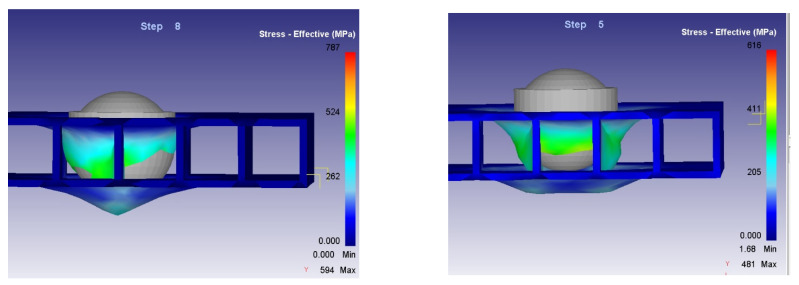
Stress-effective efforts of the structure in the two considered cases.

Figure 13 presents the stress-effective efforts for both considered cases.

Contrary to expectations, it is observed that the level of stress-effective effort in Case 2 is lower than in Case 1. It was assumed that in the second case, due to the fact that there were two vertical walls on the direction of action, which offered a higher rigidity and resistance, the level of effort would be higher. However, this can be explained by the hemispherical shape of the impactor head. In Case 1, the first point of contact is made in the direction of the partition wall which, during the whole process, is subjected to deformation, generating great efforts. In the second case, the walls are on the sides of the impactor head. Initially, the impactor head acts on the horizontal flat surface, which has a low resistance. Subsequently, after the contact between the partition walls has been cancelled, the punch will act on the partition walls.

**Figure 14:**
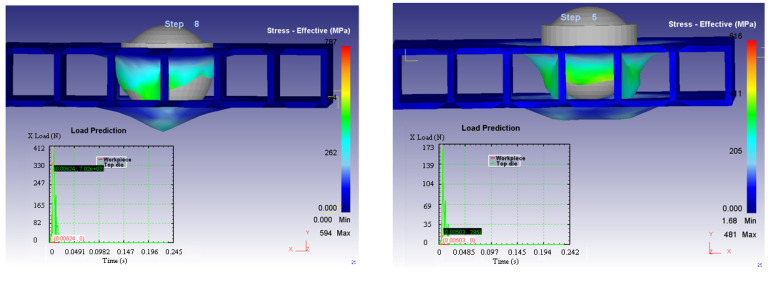
Stress-effective efforts—X axes in the two considered cases.

**Figure 15:**
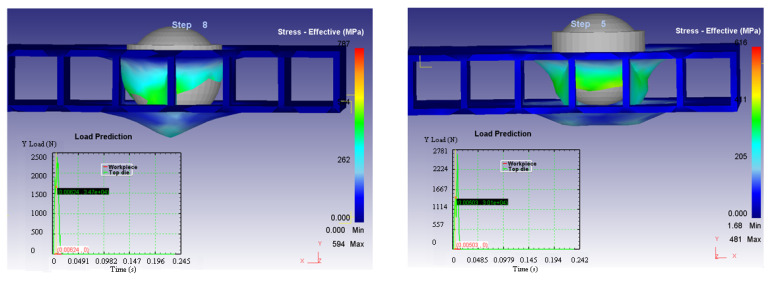
Stress-effective efforts—Y axes in the two considered cases.

**Figure 16:**
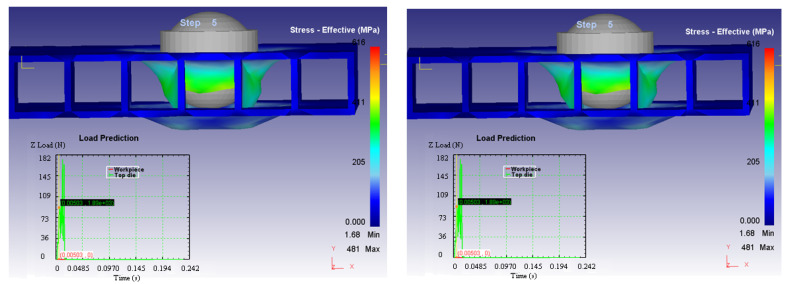
Stress-effective efforts—Z axes in the two considered cases.

Based on these considerations, it can be concluded that the arrangement of the composite structure in relation to the impactor head has an influence on: the process times (a shorter time in Case 2); the forces in the system (Case 2 requiring high values for the Y axis direction, perpendicular to the structure, but much higher in the other two directions which leads to a lower resultant force); and the variation of the deformation speed on the three axes for the two cases (presented in Figure 14, Figure 15 and Figure 16).

**Figure 17:**
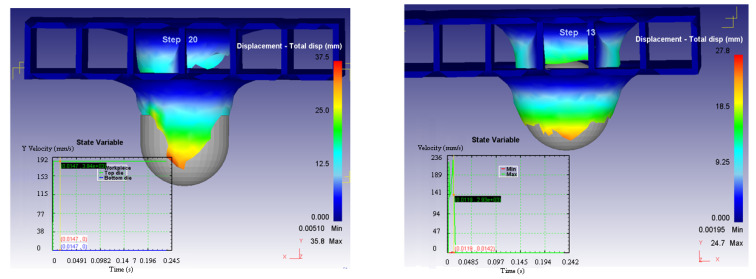
Displacement variation in the two considered cases.

Another variable that is influenced by the relative position between the impactor head and the composite structure is the size of the displacements of the structure, illustrated in Figure 17.

## 5. Conclusions

Modelling and finite element analysis are particularly important in the present research context, providing an impressive amount of information regarding the impact behavior of polymeric composites reinforced with knitted 3D sandwich fabrics. However, this information has no value if not validated by one or more experimental tests carried out in similar conditions to those under which the analysis was performed.

An FEM model was proposed in the paper, in which the ensemble knitted reinforcement and matrix is considered a continuous material with specific properties. This equivalent material was used to build the model and to simulate low velocity impact behavior.

This is a different approach than models presented in the literature as it eliminates the complex and restrictive geometrical models of the knitted structures, especially difficult and time consuming for 3D geometries and the specific connections between layers. The model was developed using Deform 3D software, calibrated using the data from an initial test carried out for all experimental variants.

The FEM model offered important information concerning the behavior structure during impact, showing how the material reacts with the impactor, how it breaks and to which level of stress. The simulation takes into consideration both situations in which the composite can be impacted during use: the impact can occur perpendicular to a connecting wall or between two connecting walls.

The behavior can be visualized for the structure as a whole and, for different sections, be considered significant. Furthermore, the structure’s strain can be visualized at any moment. This is not possible for the tests used for validation due to the very short time interval, and the impossibility to record inside the structure, as well as to record all significant stages using conventional means.

Apart from the information regarding the material deformation (that can be presented as a ‘movie’ about this event), important data are gained concerning the displacement/strain/stress/effort, as well as the deformation forces for single moments during the impact and a diagram of their evolution throughout the entire phenomenon.

## Figures and Tables

**Table 1:** Characterisation of the experimental variants (weft knitted spacer fabrics).

Fabric	Outer Layers			Connecting Layer		
	Yarn Type	Layer Structure	Yarn Linear Density (Tex)	Yarn Type	Layer Structure	Yarn Linear Density (Tex)
1	Kevlar 49^®^	Plain jersey	28	Kevlar 49^®^	Plain jersey	28
2	Kevlar 49^®^	Plain jersey	28	Linen	Plain jersey	20
3	Kevlar 49^®^	Plain jersey	28	Kevlar 49^®^	Plain jersey	28
	Twaron^®^	Plain jersey with inlay	6			
4	Kevlar 49^®^	Plain jersey	2	Linen^®^	Plain jersey	20
	Twaron^®^	Plain jersey with inlay	6			

**Table 2:** Physical mechanical characteristics of composite materials.

	Structure Variant				Polymeric Matrix	
	Kevlar–Twaron		Kevlar–Linen		Epoxy Resin	Polyester Resin
	Row	Wale	Row	Wale		
Young module [N/mm^2^]	6376	30,411	6161	5513	2900	4000
Force [N]	2430.59		745.18		-	-
Thread weight [g]	6.4		3.7		-	-
Composite sample weight [g]	19.3		14		-	-
Dimension of the impactor head [mm]	20					

**Table 3:** Working parameters.

Parameter	Value	Parameter	Value
Impact Energy [J]	37.095	Carriage Mass [kg]	4.3
Impact Velocity [m/s]	3.835	Applied Mass [kg]	0
Impact Height [mm]	750	Total mass [kg]	5.045
Impact Point Offset [mm]	0.000	Support Type	-
Extension Length [mm]	0.000	Support Diameter [mm]	20
Extension Mass [kg]	0.000		

**Table 4:** Extracted information obtained from finite element analysis.

Step No.	Mesh No.	Stroke	Time (s)	Load X (DaN)	Load Y (DaN)	Load Z (DaN)	Speed X (mm/s)	Speed Y (mm/s)	Speed Z (mm/s)	Volume (mm^3^)
−1	1	0	0	--	--	--	0	0	0	61,478.8
5	1	0.892857	0.00357142857143	4.7649	1272.9	2.4996	0	250	0	62,600.4
10	1	1.78571	0.00714285714286	0.851317	1296.5	5.0737	0	250	0	62,600.5
15	1	2.67857	0.0107142857143	2.3586	1362.9	5.6681	0	250	0	62,600.5
20	1	3.57143	0.0142857142857	1.4461	1449.8	1.5543	0	250	0	62,600.5
25	1	4.46429	0.0178571428571	2.2943	1522.6	10.955	0	250	0	62,600.5
30	1	5.35714	0.0214285714286	101.61	1604.9	103.43	0	250	0	62,600.5
35	1	6.25	0.025	2.7895	1638	25.168	0	250	0	62,600.5
40	1	7.14286	0.0285714285714	124.62	1706.6	49.233	0	250	0	62,600.5
45	1	8.03571	0.031428571429	132.49	1830.6	47.951	0	250	0	62,600.5
50	1	8.92857	0.0357142857143	160.64	1565.8	20.883	0	250	0	62,600.5
55	1	9.82143	0.0392857142857	9.905	1932.3	4.7249	0	250	0	62,600.5
60	1	10.7143	0.0428571428571	181.59	2004.4	16.246	0	250	0	62,600.5
65	1	11.6071	0.0464285714286	149.33	2032.4	2.2658	0	250	0	62,600.5
70	1	12.5	0.05	140.42	2091.5	14.438	0	250	0	62,600.4

## Data Availability

Not applicable.
